# Be like water, my cells: cell plasticity and the art of transformation

**DOI:** 10.3389/fcell.2023.1272730

**Published:** 2023-10-11

**Authors:** Patrizia Cammareri, Kevin B. Myant

**Affiliations:** Cancer Research UK Scotland Centre, Institute of Genetics and Cancer, The University of Edinburgh, Edinburgh, United Kingdom

**Keywords:** cell plasticity, cancer, metaplasia, EMT, therapy

## Abstract

Cellular plasticity defines the capacity of cells to adopt distinct identities during development, tissue homeostasis and regeneration. Dynamic fluctuations between different states, within or across lineages, are regulated by changes in chromatin accessibility and in gene expression. When deregulated, cellular plasticity can contribute to cancer initiation and progression. Cancer cells are remarkably plastic which contributes to phenotypic and functional heterogeneity within tumours as well as resistance to targeted therapies. It is for these reasons that the scientific community has become increasingly interested in understanding the molecular mechanisms governing cancer cell plasticity. The purpose of this mini-review is to discuss different examples of cellular plasticity associated with metaplasia and epithelial-mesenchymal transition with a focus on therapy resistance.

## Introduction

In 1957, the biologist Conrad Waddington described embryonic development as a series of distinct paths that stem cells follow before reaching an irreversible terminal differentiation stage (Waddington, 2014; Rajagopal and Stanger, 2016). This predictive model introduced the concept of unidirectional hierarchical cell fate. However, the notion of irreversible cellular commitment, meaning a cell becomes a fixed type of specialized cell with a determined physiological function, has been radically revised during the late 20th and early 21st centuries. John B. Gurdon challenged Waddington’s dogma by transferring the nucleus of fully differentiated adult cells into an enucleated eggs which results into fully developed adult animal (Gurdon, 1962). Later, Shinya Yamanaka, by introducing four transcription factors (Klf4, c-Myc, Oct3/4, and Sox2), reprogrammed fully differentiated mouse fibroblast cells into a pluripotent stem cell state, capable of producing multiple cell types (Takahashi et al., 2007). Gurdon and Yamanaka’s results revolutionized the notion of terminal cell differentiation, relaunched the concept of cell plasticity and in 2012 they were awarded the Nobel Prize for Physiology or Medicine for their ground-breaking discoveries.

Since then, a growing number of studies conducted on different organs have demonstrated the ability of committed cells to be reprogrammed in response to various stimuli. Currently the term “plasticity” refers to the cell’s ability to adopt different identities through multiple mechanisms, such as dedifferentiation or transdifferentiation, particularly when tissue homeostasis is perturbed. Interestingly, cancer cells can alter their identity by adopting alternative developmental lineages. Lineage plasticity is a mechanism commonly employed by tumour cells to evade chemotherapy treatment, leading to intra-tumour heterogeneity, therapy resistance and metastasis. The development of single cell sequencing technologies has profoundly contributed to gathering new insight into tumour cell plasticity. This has led to the identification of previously uncharacterized cell states and allowed us to trace transitions between different states. As a result, we have gained a better understanding of the mechanisms underlying tumour heterogeneity and resistance to therapy offering us new therapeutic prospects.

Here, we will discuss concisely recent literature on metaplasia and EMT, two biological processes that regulate the emergence of distinct cell identities in response to specific stimuli, with a final emphasis on lineage plasticity and therapy resistance.

## Metaplasia

Metaplasia is an adaptive process characterized by the replacement of a differentiated cell type with another cell type not normally observed in the tissue where it occurs. It is mainly characterized by changes in cell morphology and expression of lineage-specific markers, including transcription factors defining tissue-specific identity. Metaplasia affects organs constantly exposed to external damaging agents or suffering from chronic inflammation, such as the lungs, intestinal tract, and pancreas. It is generally considered a protective process to limit tissue injury. However, metaplasia is also considered a precursor to dysplasia, and can be associated with an increased risk of malignancy. Here, we will discuss the most common forms of inflammatory-driven metaplasia which includes the specialized intestinal metaplasia (SIM) reported in Barrett’s esophagus (BE) and the acinar-ductal metaplasia (ADM) observed in the pancreas.

**FIGURE 1:**
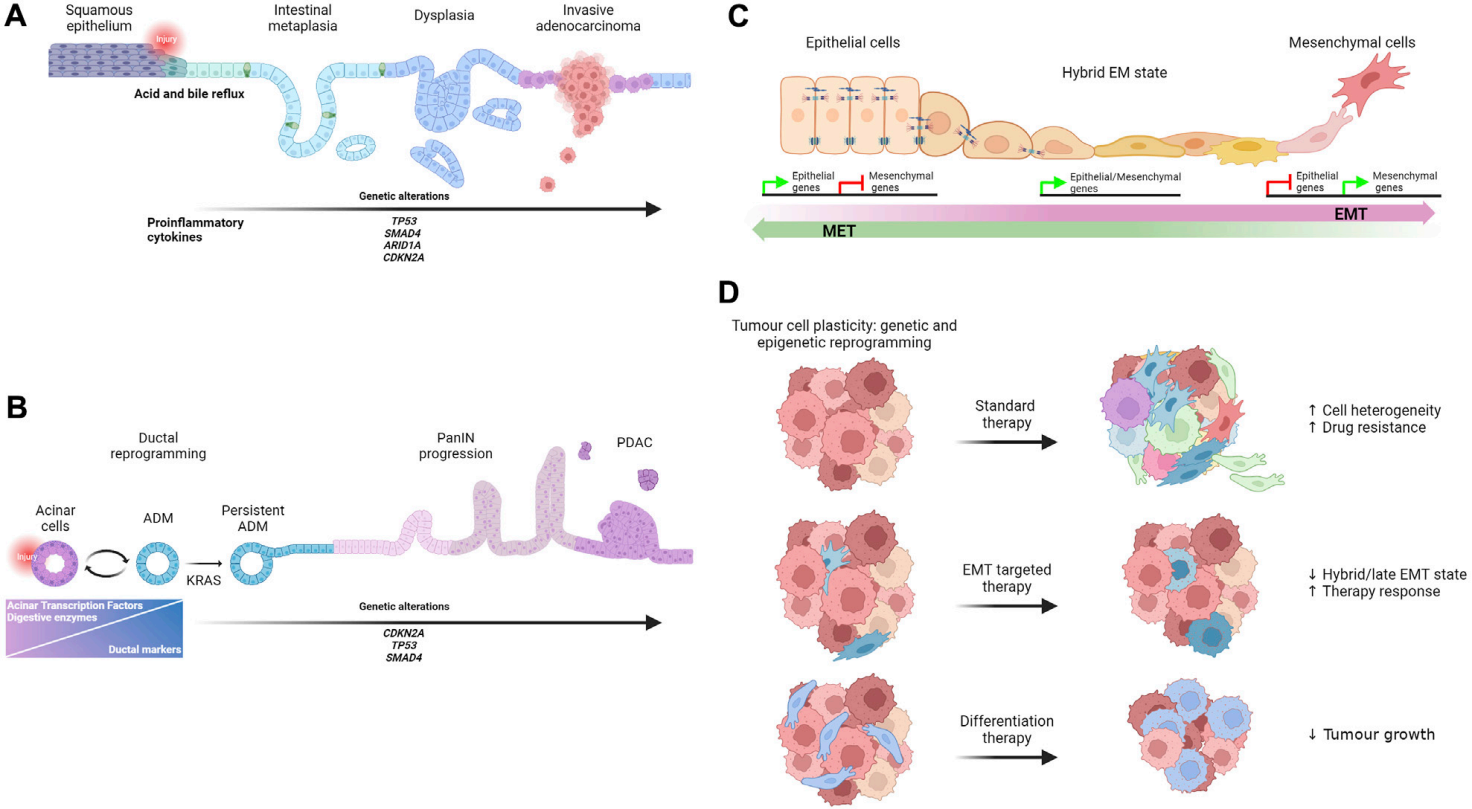
**(A)** Schematic representation of BE initiation and progression to adenocarcinoma. **(B)** Model of ADM origin and progression to PDAC. **(C)** Illustration of morphological changes that occur during the process of EMT as well as during the reverse process. **(D)** Schematic overview of drug response in cancer.

BE is a metaplastic condition in which the normal stratified squamous epithelium, covering the distal esophagus, is gradually replaced with a columnar intestinal-like epithelium, including goblet cells, in response to chronic exposure to acid and bile in patients with gastroesophageal reflux disease (GERD). It has been suggested that the goblet cells’ cytoprotective role makes the epithelium more resistant to GERD-induced damage. Nevertheless, BE can progress into low-grade and high-grade dysplasia and culminate in the development of esophageal adenocarcinoma (EAC) (Figure 1A). BE pathogenesis has been largely debated and several cell types have been proposed as cells of origin (Souza and Spechler, 2022). However, the lack of an *in vivo* model able to recapitulate many aspects of human disease has left the question about its origin and the association to EAC unanswered for several years. In 2012, M. Quante et al. (2012) using lineage tracing experiments in a mouse model of chronic esophageal inflammation, identified Lgr5^+ve^ progenitor cells in the gastric cardia as the origin of BE. In 2017, M. Jiang et al. (2017) demonstrated that p63^+ve^, KRT5^+ve^, KRT7^+ve^ basal progenitor cells, localized in the squamous/columnar junction of the upper gastrointestinal tract, can differentiate into intestinal cells, including goblet cells, recapitulating SIM. Few years later, Nowicki-Osuch et al. (2021) conducted comparative multi-omics analyses on different potential tissues of origin, including the fresh isolated esophageal submucosal glands, and they found that, to repair GERD-damaged squamous epithelium, undifferentiated cells at the gastric cardia can adopt a new identity resulting in SIM. Additionally, the authors report that the undifferentiated phenotype observed in BE was maintained during EAC transition, even when BE was no longer detectable (Nowicki-Osuch et al., 2021). Although these findings are mainly correlative, they support what other groups have previously hypothesized that BE originates from the gastric cardia lineage (Quante et al., 2012) and that it can be considered a precancerous condition.

Independently of the cell of origin, what remarkably emerges from several studies is the contribution to the BE reprogramming process of key regulators of intestinal epithelial identity, such as CDX2 and HNF4α (Jiang et al., 2017; Nowicki-Osuch et al., 2021) which are both upregulated in BE, along with other intestinal TFs (Rogerson et al., 2019). In addition, ectopic expression of CDX2 leads to intestinal metaplasia in the mouse gastric mucosa (Silberg et al., 2002) and HNF4a-mediated CDX2 expression intestinalize antral stomach organoids by regulating chromatin access at specific intestinal enhancers suggesting that enhancer accessibility can serve as mechanism for protecting the identity of the tissue of origin (Singh et al., 2022). Both CDX2 and HNF4α are TFs essential for embryonic intestinal development and adult intestinal homeostasis and whose deregulation impairs cell identity (Garrison et al., 2006; Hryniuk et al., 2012; San Roman et al., 2015). For example, the conditional deletion of *Cdx2* from early endoderm affects the expression of additional TFs that define the intestinal identity, such as CDX1 and HNF1α, and leads to squamous differentiation through the activation of the esophageal differentiation program (Gao et al., 2009). Therefore, a better understanding of how specific TFs regulate esophageal cell identity and reprogramming is important for better defining the molecular mechanisms that cause BE and the transition to EAC.

ADM is a reversible and highly regulated form of cell plasticity induced by the inflammatory microenvironment and it is frequently observed in the pancreas affected by acute or chronic pancreatitis. Pancreatic acinar cells are one the major cell components in the pancreas. They are postmitotic cells responsible for synthesising, storing and secreting digestive enzymes, such as amylase and lipases. Interestingly, pancreatic acinar cells are sensitive to different stress conditions. In response to inflammation or injury, pancreatic acinar cells can dedifferentiate into embryonic progenitor cells with a ductal-like phenotype. Specifically, during ADM, acinar cells reduce the expression of acinar-specific TFs, such as MIST1, PTF1A, GATA 6, and NR5A2, and they rapidly decrease the expression of digestive enzymes that could enhance the inflammatory response (Zhu et al., 2004; Krah et al., 2015; Martinelli et al., 2013; von Figura et al., 2014; Pinho et al., 2011). These changes are accompanied by the expression of ductal markers, such as KRT19, osteopontin and TFs expressed by pancreatic embryonic progenitors such as Sox9 (Kopp et al., 2012; Prevot et al., 2012; Mills and Sansom, 2015). The resulting hybrid ductal-like cells are proliferative and able to replace damaged acinar cells contributing to regeneration after injury. Therefore, ADM is considered a protective and reversible repair mechanism that pancreatic cells adopt to limit the damage and reconstitute tissue integrity. However, in the presence of persistent inflammation, deregulated signalling pathways and KRAS oncogenic mutations, ADM becomes persistent and can progress to different grades of pancreatic intraepithelial neoplasia (PanIN) lesions (Habbe et al., 2008) and ultimately to pancreatic ductal adenocarcinoma (Ji et al., 2009; Guerra et al., 2011; Ardito et al., 2012; Bailey et al., 2016a; Bailey et al., 2016b; Storz, 2017) (Figure 1B). *Kras* mutant cells acquire multiple highly plastic states defined by distinct chromatin accessibility profiles (Burdziak et al., 2023). The combination of *Kras* mutations and injury-induced inflammation is associated with a variety of gene expression programs, some of which are maintained in PDAC, suggesting that the combined effect of early genetic mutations and tissue injury primes cells for tumour development (Alonso-Curbelo et al., 2021; Burdziak et al., 2023). Based on these studies, it appears that the switch of ADM from a protective mechanism to a precancerous lesion is a critical step that require particular attention. Recent intriguing data generated by Andrea Viale’s laboratory demonstrate that a transient inflammatory event induces an adaptive response characterized by persistent transcriptional and epigenetic changes that facilitate reacquiring ADM, if a second inflammatory challenging event occurs, thereby providing a powerful protective mechanism (Del Poggetto et al., 2021). However, the early onset of oncogenic Kras activation can override the effect of this initial defence mechanism contributing actively to the development of tumours. Thus, one can speculate that a controlled induction and/or stabilization of ADM state could be beneficial for pancreatitis treatment, and it may prove to be an effective strategy to prevent patients from progressing into PDAC.

This could be achieved by pharmacologically intervening in key pathways regulating the initiation and progression of ADM.

As suggested by Del Poggetto et al. (2021) treatment with MAPK pathway agonists could reduce the selective pressure to activate oncogenic Kras representing a strategy to induce ADM and ameliorate pancreatitis. Although this strategy may prove to be an initial effective treatment option, the long term consequences of the MAPK pathway and inflammation should be carefully investigated to exclude the possibility of induction of a neoplastic commitment, as observed with Kras mutations (Alonso-Curbelo et al., 2021). Inhibition of Numb could represent another beneficial therapeutic option. Loss of Numb induces progression to ADM but reduces the proliferation of PanIN, thus blocking the progression to tumour (Greer et al., 2013; Mills and Sansom, 2015). A recent study proposes a more complex cell heterogeneity in injury-induced ADM. Lineage tracing and single-cell RNA sequencing (scRNA seq) analysis of ADM identified acinar cells undergoing a pyloric-type metaplasia and transitioning to mucinous progenitor cell-like populations that differentiate in multiple subtypes of tuft and enteroendocrine cells (Ma et al., 2022). Overall, each of the different cell types identified in ADM may be susceptible to oncogenic transformations; therefore, a better molecular characterization of the cell types and cell states contributing to the unresolved ADM will help to identify new therapeutic targets to block the progression to PDAC.

## Epithelial-mesenchymal transition

EMT is a transient physiological process essential for embryonic development and tissue formation. During EMT epithelial cells transit to cells with a complete mesenchymal phenotype through a dynamic program regulated by the expression of a core set of EMT transcription factors (EMT-TFs) such as Twist1, Zeb1, Snail1, and Slug (Thiery et al., 2009; Nieto, 2011) (Figure 1C). Despite years of debate about the presence and function of EMT in adults, the reactivation of the EMT program has been reported in several pathological conditions including many metastatic cancer types. Accordingly, the conditional knockout of Zeb1 in a metastatic pancreatic cancer mouse model fix the cells in an epithelial state and strongly reduce the progression to metastatic tumours (Krebs et al., 2017). However, Snail1 or Twist1 depletion, in the same tumorigenic mouse model, has no effect on metastasis formation (Zheng et al., 2015). Although these findings confirm the tissue specific function of EMT-TFs already proposed by other groups (Caramel et al., 2013; Ye et al., 2015), one of the major caveats is the use of the mesenchymal marker a-smooth muscle actin as an indicator of EMT, despite its expression being rarely detected in the same mouse model. In addition, the compensatory effects of key EMT-TFs, such as Zeb1 and Slug, expressed in the Twist and Snail knockout, cannot be excluded (Aiello et al., 2017). An alternative explanation that could explain the apparent lack of EMT requirement for metastasis in Zheng’s paper may come from studies that focus on the dynamic activation of specific EMT-TFs. Only the transient, and not continuous, expression of Twist1 induces a novel cell state that promotes invasive growth in a model of immortalized human mammary epithelial cells (Schmidt et al., 2015). These results are consistent with previous published data showing that Snail1 and Twist1 transient expression leads to metastatic growth in mouse models of breast and squamous cell carcinomas (Tsai et al., 2012; Tran et al., 2014). Due to the transient nature of EMT during tumorigenesis, tracing the dynamic of this process has been challenging. In 2020, using dual recombinases-mediated genetic lineage tracing (Cre/Lox and Dre/Lox), Li Yan and colleagues detected EMT activity in a spontaneous breast-to-lung tumour metastasis model. In this model, cells start expressing the EMT marker N-cadherin during the early phases of dissemination and then form the majority of lung metastases. Consistently, N-cadherin deletion significantly reduces the number of metastases compared to control mice (L et al., 2020; Vieugue and Blanpain, 2020). It has now been widely demonstrated that, rather than transitioning to a full mesenchymal state, cancer cells undergo a partial or “hybrid” EMT showing both epithelial and mesenchymal markers being expressed by the same cancer cell, including circulating tumour cells. The presence of a partially activated EMT program is sufficient to provide cancer cells with highly invasive and metastatic potential (Yu et al., 2013; Ruscetti et al., 2015; Puram et al., 2017). In 2021, Luond et al. (2021) using a novel tamoxifen-inducible dual recombinase lineage tracing approach, were able to map partial and full EMT states in a mouse model of metastatic breast cancer. They showed that mammary cancer cells rarely reach full EMT, but once established, cells tend to retain this phenotype. Conversely, cells undergoing partial EMT frequently go through the inverse process called mesenchymal-epithelial transition (MET).

Various degrees of dynamic hybrid states have been reported among cancer cells, indicating their ability to shift between multiple states. Compelling work by I. Pastushenko and co-workers, performed on a mouse model of skin squamous cell carcinomas undergoing spontaneous EMT and on metaplastic-like mammary tumours, led to the identification of different tumour subpopulations with a spectrum of intermediate EMT states which contribute to tumour heterogeneity. Interestingly, the different intermediate subpopulations show varying degrees of invasive and metastatic capacity, with hybrid epithelial-mesenchymal cells showing an increased capacity for entering the circulation and forming lung metastases (Pastushenko et al., 2018). A number of significant papers have been published since these data were first reported by the Blanpain’s group. These studies demonstrate that multiple EMT states are present in a variety of different models including glioblastoma (Neftel et al., 2019) and melanoma (Wouters et al., 2020).

Intriguing discussion has arisen regarding the existence of a phenotypic continuum rather than the presence of stable discrete EMT states. MAGIC, a computational method that recovers missing gene expression in individual cells, has significantly improved scRNA seq quality data and, when applied to an EMT model, has revealed a transcriptional continuum gradient with the majority of cells residing in intermediate states (van Dijk et al., 2018). This alternative EMT model has been confirmed by other groups analysing epithelial cells undergoing spontaneous EMT or following treatment with TGFβ (McFaline-Figueroa et al., 2019). In 2021, Simeonov et al. (2021) developed an excellent CRISPR/Cas9-based tool capable of capturing transcriptional and phylogenetic information at a single cell level. This method, called macsGESTALT, was applied to a model of pancreatic cancer metastasis. Their findings support the notion that a continuum of EMT states exists *in vivo,* where cells gradually lose epithelial markers and gradually acquire EMT markers with late hybrid EMT transcriptional signatures associated with higher risk of death in patients with PDAC and lung cancer (Simeonov et al., 2021). The gradual changes in gene expression observed within a tumour translates in a gradient of tissue morphologies. Multiplexed 3-dimensional analysis in combination with spatial transcriptomic analysis has highlighted the continuous phenotypic changes that occur from the centre of the tumour toward the invasive front and in the tissue microenvironment in colorectal cancer samples (Lin et al., 2023; Messal and van Rheenen, 2023).

Finally, it is worth mentioning that alternative EMT programmes can be adopted during tumour progression. Using a lineage-labelled murine model of ductal pancreatic cancer, Aiello et al. (2018) described an additional EMT model that involve loss of the epithelial phenotype through protein internalization, rather than the transcriptional repression commonly associated with the classical EMT phenotype. Interestingly, this alternative post translational-regulated program is mainly associated to collective cell migration with cell maintaining cell-cell contacts during the metastatic process. Overall, despite the initial controversy about EMT, the experimental evidence published in recent years have strongly supported its critical role in cancer, regardless of the mode of action.

## Lineage plasticity and drug resistance

During cancer progression, cancer cells undergo extensive genetic and epigenetic reprogramming which reveals their latent ability to change lineages. The expansion of emerging subclones with a new identity is highly context specific, can contribute to intra-tumour cell heterogeneity and adversely affect treatment response as new cell types have different sensitivity to the original drug target, which may even be no longer present. Thus, targeting communal regulatory mechanisms of the epithelial-mesenchymal plasticity could prove to be a more efficient therapeutic strategy (Cook and Vanderhyden, 2022) (Figure 1D). Several studies report a link between EMT and the development of drug resistance in different *in vitro* and *in vivo* tumour models. For instance, EMT has been associated with resistance to gemcitabine treatment in models of PDACs (Shah et al., 2007; Wang et al., 2009). Accordingly, loss of Snail and Twist1 enhanced sensitivity to gemcitabine by regulating the expression of nucleosides transporters and resulting in an increased overall survival (Zheng et al., 2015). Furthermore, EMT cells appear to be resistant to cyclophosphamide, one of the most used chemotherapeutic agents for treating breast cancer. It is important to note that surviving EMT cells significantly contribute to lung metastasis formation (Fischer et al., 2015). Debaugnies et al. (2023) identified RHOJ, a small Rho GTPase of the Cdc42 subfamily, as regulator of anti-cancer therapy resistance in a mouse model of skin squamous cell carcinoma undergoing spontaneous EMT. Mechanistically, high level of RHOJ in the EMT-resistant cells prevents the accumulation of DNA damage and alleviates the replicative stress by promoting the activation of “dormant origin” of DNA replication. This process facilitates the cell’s ability to repair DNA damage, which allows them to survive treatment (Debaugnies et al., 2023). More recently, promising data published by Lengrand et al. (2023) show that the pharmacological inhibition of netrin-1, a protein expressed in EMT tumour cells, increases the proportion of epithelial cells, while reducing the proportion of hybrid and late EMT cells. Importantly this sensitizes cells to chemotherapy treatment. This might represent a novel therapeutic option for targeting EMT (Lengrand et al., 2023).

The process of therapy resistance is further complicated because chemotherapy treatment itself can trigger lineage plasticity and promotes drug resistance. As an example, EGFR mutant lung adenocarcinomas that are repeatedly treated with tyrosine kinase inhibitors develop resistance within months of treatment. In a subgroup of patients with resistance to treatment, evidence of small-cell lung cancer transition has been reported, indicating that treatment can trigger the transformation of the tumour into a distinct histological subtype (Oser et al., 2015). Transdifferentiation as mechanism of drug resistance has been also reported in a mouse model of castration resistant prostate cancer (CRPC) treated with the anti-androgen abiraterone (Zou et al., 2017). Similar histologic features, indicative of CRPC becoming an aggressive neuroendocrine type, has been observed in patients resistant to androgen receptor inhibitor treatment (Beltran et al., 2011; Beltran et al., 2016). Lineage plasticity in response to treatment has been also reported in patients with metastatic muscle invasive bladder cancer (MIBC). In particular, partial squamous differentiation has been observed in the basal cells of MIBC following chemotherapy. Combination of ATAC-sequencing and proteomic analyses indicates the lysosomal cysteine proteinase Cathepsin H (CSTH) as potential regulator of this distinctive form of lineage plasticity. Gradual increase of CSTH has been observed during this partial basal-squamous transition. An intriguing finding in this study is that treatment with the CSTH inhibitor, E64, caused full squamous differentiation and tumour growth suppression indicating that differentiation therapy could be a potential treatment alternative for patients suffering from MIBC (Wang et al., 2022). The use of differentiation therapy to leverage lineage plasticity is an innovative classical approach that may be applied to treat solid tumours in which cell plasticity contributes to chemotherapy resistance. Thus, a better understanding of the molecular changes that occur in multiple resistant clones, which emerge following therapy treatment, is essential for the identification of targets that can be exploited by differentiation therapy. Recently, Goyal et al. (2023) developed a novel approach, called FateMap, to follow the fates of resistant clones. Using a combination of DNA barcoding, scRNA seq and computational analysis, the authors show that the transcriptional and phenotypic variability in the resistant clones is determined prior to drug treatment and it is not dictated by genetic mutations. These findings suggest that the characterization of the initial intrinsic molecular state of a tumour cell is critical to predict its fate following drug administration. This is essential for the development of new therapeutic approaches and to prevent relapse in patients.

## Conclusion

The studies discussed in this mini-review highlight the cells’ remarkable capacity to adapt and change their fate and identity in response to stimuli and cancer. Perturbations of adult tissue integrity result in a prompt adaptive response characterized by extensive cellular reprogramming. This is mainly regulated by a complex network of TFs that define cell identity. In this context, cell plasticity may serve as a repair mechanism, which, at least initially, may be beneficial since reprogrammed cells can adopt new functions to limit damage. However, it is now widely accepted that even this form of “advantageous” cell plasticity can predispose to cancer. Therefore, defining the molecular mechanisms that govern this functional switch is imperative to stop tumour progression. Considering this, several questions remain open including: how do specific insults, like, for example, inflammation, regulate the expression of TFs that determine cell identity? What are the long-term consequences of early changes in chromatin accessibility for tumour initiation and progression?

In regard to EMT, as discussed in this mini-review, the transition from an epithelial to a mesenchymal state can manifest through two distinct models: a continuum spectrum of transitional states or multiple discrete states. Despite this apparent discrepancy, perhaps caused by different technical approaches, what overall emerges from these studies is the extraordinary capacity of EMT cells to dynamically shift between multiple different states. However, in response to specific cell intrinsic and extrinsic factors, cells within the EMT continuum can stall and reside in intermediate and more stable states. This considerably contributes to tumour heterogeneity and therapy resistance. Open questions that arise from these observations are: which model plays a more critical role in tumour development and metastasis formation? Can we target a stable discrete state or a range of multiple states? Can we shift EMT cells toward a less aggressive or more targetable state?

In addition to EMT, distinct forms of cell plasticity have been reported in cancer posing new challenges in investigating cell heterogeneity and therapeutic approaches. However, given the rapid progress with single-cell sequencing technologies and methods of analysis the function of novel cell plasticity regulators will be described soon potentially unveiling new therapeutic avenues.
